# Exploring Patient Beliefs and Medication Adherence in the Mediterranean Context: A Cross-Sectional Study in Patients with Cardiovascular Diseases and Cardiometabolic Disorders in Greece—The IACT-Study

**DOI:** 10.3390/life13091880

**Published:** 2023-09-07

**Authors:** Vasiliki Belitsi, Thomas Tsiampalis, Matina Kouvari, Vasiliki Kalantzi, Odysseas Androutsos, Fotini Bonoti, Demosthenes B. Panagiotakos, Rena I. Kosti

**Affiliations:** 1 Department of Nutrition and Dietetics, School of Physical Education, Sports and Dietetics, University of Thessaly, 42132 Trikala, Greece; vbelitsi@uth.gr (V.B.); ttsiampalis@uth.gr (T.T.); vkalantzi@uth.gr (V.K.); oandroutsos@uth.gr (O.A.); fbonoti@uth.gr (F.B.); 2 Department of Nutrition and Dietetics, School of Health Science and Education, Harokopio University, 17676 Athens, Greece; mkouvari@hua.gr (M.K.); dbpanag@hua.gr (D.B.P.); 3 Faculty of Health, University of Canberra, Canberra, ACT 2617, Australia

**Keywords:** medication adherence, heart disease, cardiometabolic disorders, beliefs, views, treatment, patient-centered care

## Abstract

Background: Evidence has shown that poor adherence to vascular medications contributes to a considerable proportion of all cardiovascular disease (CVD) events and mortality. The aim of the present work was to examine patients’ beliefs/views that affect their level of adherence to the assigned medical treatment in the context of a multi-center study in Greece. Methods: Between July 2022 and April 2023, 1988 patients (1180 females) with established cardiovascular disease or relevant cardiometabolic disorders were chosen from seven medical centers in Greece. The 4-item Morisky Medication Adherence Questionnaire gauged medication adherence and investigated patients’ beliefs/views regarding treatment. Results: Among participants, 51.2% showed perfect medication adherence, contrasting with 48.8% displaying poor adherence. Patients with negative medication beliefs were around three times more likely to be non-adherent (OR = 2.73; 95% CI = 2.28–3.28). Non-adherers held concerns about drug efficacy (OR = 2.34; 95% CI = 1.10–4.97) and favored alternative therapies (OR = 2.25; 95% CI = 1.75–2.91). Conclusion: The findings highlight the significance of addressing patient beliefs/views to improve medication adherence. The distinct Mediterranean context, influenced by cultural, socioeconomic, and clinical factors, emphasizes the need for tailored interventions. This underscores the call for contextually sensitive strategies to boost medication adherence and improve health outcomes in this unique region.

## 1. Introduction

Cardiovascular diseases place a substantial economic burden on healthcare systems, individuals, and society as a whole [1,2,3,4]. In addition, metabolic dysfunctions, such as obesity and diabetes, significantly increase the risk of developing other chronic conditions (e.g., cancer, and respiratory disorders), which further burdens public health [5]. Hence, ensuring the effective treatment and management of these diseases is considered paramount. However, they often go undiagnosed and untreated, leading to a higher incidence of cardiovascular mortality [6]. Medication adherence has been defined as the extent to which a patient takes medications as prescribed by their healthcare providers [7]. Despite the availability of effective medications for cardiovascular disease (CVD) and relevant disorders, medication nonadherence remains pervasive globally. A meta-analysis that included about 2 million patients showed that only 60% of patients were adherent to their cardiovascular medications. Additionally, compared with those with good adherence, the risk of cardiovascular events or mortality in those with poor adherence increased by 20% or 35%, respectively [8]. Additionally, evidence from real-world data has shown poor adherence to vascular medications significantly contributes to suboptimal secondary care [9].

Nonadherence behaviors can be broadly categorized as either unintended, resulting from forgetfulness, visual impairments, or mobility challenges [10], or intentional, where patients deliberately avoid taking medication despite being capable of doing so [11]. At the same time, it is widely acknowledged that medication adherence constitutes a multifactorial phenomenon that is influenced by various factors, encompassing medication-related aspects, patient- and physician-related factors, as well as system-based influences [12,13,14,15,16,17,18]. In addition, patients’ adherence to medication is notably influenced by their beliefs [19] as well as by affordability issues [20]. Various studies have indicated that individuals with lower health literacy tend to harbor concerns about the potential harm of medications, resulting in under-usage and, consequently, less favorable clinical outcomes [21,22,23]. Hitherto, many studies have focused on overall negative treatment beliefs; however, there is a need for more research that delves into specific types of negative beliefs. Understanding the nuances of different negative beliefs, such as fear of side effects, skepticism about treatment efficacy, or concerns about medication cost, can provide more targeted insights for interventions.

At the same time, the Mediterranean region is recognized for its distinctive cultural, social, and economic elements that can shape patients’ healthcare beliefs, views, and adherence behaviors. In particular, the Mediterranean region stands out for its unique blend of cultural, social, and economic factors that significantly influence the way individuals approach their healthcare [24]. With its rich history and diverse societies, this region’s distinct characteristics have a direct impact on patients’ views and behaviors related to their health [25]. Recent research findings underscore the unique cultural attributes of Mediterranean societies, which are characterized by both independent and interdependent cultural elements. These attributes distinguish them significantly from Eastern and Western societies [26]. Furthermore, scholarly investigations have highlighted the influential role of cultural norms in shaping societal perspectives on health beliefs and behaviors [27].

From centuries-old traditions to modern influences, the Mediterranean context molds how people view medical treatments, adhere to prescribed medications, and engage with healthcare providers [28]. More precisely, the Mediterranean region’s cultural values, emphasizing close-knit communities and familial bonds, significantly shape patients’ views and actions toward medical treatments [29]. This emphasis on interpersonal relationships can lead to collective family decision-making about healthcare, often influenced by family consensus. Furthermore, the Mediterranean lifestyle, characterized by routine physical activity and stress-relief practices, promotes a holistic health perspective that could contribute to more extensive adherence behaviors [30]. Nonetheless, economic considerations, including diverse healthcare access, also play a role, potentially impacting treatment affordability, accessibility, and ultimately medication adherence. Throughout history, Mediterranean societies have placed their trust in a variety of plants and herbs, recognizing their potential healing qualities. This reliance on botanical remedies is deeply embedded in traditional wisdom and cultural convictions regarding the curative attributes of specific flora. These include medicinal herbs and plants employed to address an array of health concerns, encompassing the treatment of everyday maladies, the management of persistent conditions, and the enhancement of overall health [31]. It is worth emphasizing that the utilization of medicinal herbs is extensive across the Mediterranean, although its prevalence fluctuates between nations and locales within the region. Each culture within the Mediterranean boasts a distinct repertoire of age-old remedies and herbal customs.

Therefore, this cross-sectional study seeks to shed light on the beliefs of patients with established CVD or cardiometabolic disorders that affect their level of adherence to medication. While existing literature has addressed similar themes in diverse settings, the unique cultural, social, and economic factors inherent to the Mediterranean region necessitate localized investigations. The specific objectives of this study were: (a) to record the beliefs of patients regarding medication; and (b) to investigate the moderating effect of demographic and clinical factors on the association of the reported beliefs/views with patients’ level of adherence to medication.

The innovation of our study lies in its exploration of patient beliefs within the context of established CVDs and cardiometabolic disorders, uncovering their influence on medication adherence. Although previous literature has touched on similar topics in various contexts, what sets our study apart is its tailored investigation into the Mediterranean region’s distinctive cultural, social, and economic factors. These regional nuances necessitate a localized approach to understanding patient beliefs and adherence behaviors. Specifically, our study uniquely aims to not only document patients’ medication-related beliefs but also scrutinize how demographic and clinical factors interact with these beliefs to influence adherence levels. This comprehensive analysis provides novel insights into the intricate interplay between patient beliefs and medication adherence within the Mediterranean setting, contributing to the development of more contextually informed interventions for improved treatment adherence and health outcomes.

## 2. Materials and Methods

### 2.1. Study Design and Scope

This is a cross-sectional observational study conducted in 2022–2023. The study aimed to investigate the prevalence and types of treatment beliefs among patients diagnosed with CVD or relevant cardiometabolic disorders and explore their association with medication adherence.

### 2.2. Setting

The study took place in the 7 health administrative regions of Greece (Attica, Piraeus, and Aegean; Macedonia, Macedonia, and Thrace; Thessaly and Central Greece; Peloponnese; Ionian Islands; Epirus and Western Greece; Crete); and the enrolment procedure was carried out during 2022–2023. Except these regions, participants were also enrolled in the study from the following medical centers: the 1st Multipurpose Municipal Clinic of Solonos Athens, the 2nd Multipurpose Municipal Clinic of Neos Kosmos, the 3rd Municipal Clinic of Petralona, the 4th Municipal Clinic of Kolonou, the 6th Multipurpose Municipal Clinic of Kypseli, Trikala-Farkadona Medical Center, Pyli Medical Center, and Kalambaka Medical Center.

### 2.3. Sample

A total of 1988 patients with CVD and/or other cardiometabolic disorders or risk factors (1180 females) aged on average 64 years old (mean (SD): 63.9 (13.4) years) were enrolled in this cross-sectional study from the aforementioned regions. A systematic sampling approach was employed, with consecutive recruitment of participants meeting the eligibility criteria until the target sample size was attained. Ethical considerations and practical constraints were considered when finalizing the sample size. The selected sample encompasses a diverse group of patients, offering a comprehensive overview of treatment beliefs and adherence patterns within this population.

### 2.4. Eligibility Criteria

Participants eligible for this cross-sectional study were recruited from the specified health regions and medical centers between July 2022 and April 2023. The following eligibility criteria were utilized to identify potential participants:

Age: Participants aged 18 years and older were included in this study.

Diagnosis: This study considered individuals diagnosed with CVD (i.e., coronary heart disease, stroke) or other cardiometabolic conditions, including hypertension, type 2 diabetes, type 1 diabetes, hypercholesterolemia, elevated triglycerides, obesity, and non-alcoholic fatty liver disease, as eligible for participation.

Treatment Regimen: Eligibility was extended to participants who had been prescribed one or more medications for cardiometabolic disorders for a minimum duration of 1 year.

### 2.5. Bioethics

Prior to commencing this study, the researchers sought approval from the relevant department of the Greek Ministry of Health and adhered to the principles outlined in the Declaration of Helsinki. In addition, this study was implemented in accordance with the ethical standards of the University of Thessaly Ethics Committee (Ethics 11-14/07/2022). The research objectives and procedures were conveyed to all individuals involved, and patients provided written consent before participating in this study.

### 2.6. Measurements

The IAATQ-CMD questionnaire, which was found to be both reliable and repeatable [30], comprised inquiries concerning demographic and behavioral attributes, a comprehensive medical history covering cardiovascular risk factors, and the dietary and lifestyle habits of the participants. Additionally, it encompassed questions related to the therapeutic protocol and the patient’s beliefs about it [32].

### 2.7. Socio-Demographic Characteristics

The educational level of participants was assessed based on the highest level of education attained, which included options such as Primary school, Gymnasium graduate, High school graduate, Graduated from Technical High School, TEI graduate, University graduate, Postgraduate, PhD, and Others. For simplicity, participants were categorized into three groups: Group I for Primary school, Group II for Secondary education, and Group III for higher tertiary education.

The occupational status of participants was also recorded, encompassing options such as State employee, Private employee, Freelance, Retired, Unemployed, Student, and Other. Based on this information, patients were further divided into the following groups: Group I for Employed/Freelance and Group II for Unemployed/Retired. Moreover, the mean individual/family annual income was documented, and participants’ financial status was classified into two groups: low (income < 18,000 euros/year) and at least moderate (income ≥ 18,000 euros/year). The specific cut-off point for the annual family/ income, as according to the OECD, is that in Greece, the average household net-adjusted disposable income per capita equals approximately 18,000 euros/year. Finally, information regarding marital status (Single, Married, Widowed, Divorced, Cohabitation, Single without cohabitation), age (in years), sex (Male/Female/Non-Binary), and nationality (Greek/Other) was also collected.

### 2.8. Anthropometric Characteristics

Patients’ weight and height were self- reported, and based on these, body mass index (BMI) was calculated by dividing the weight (in kilograms) by the standing height (in meters squared), and according to standard guidelines, obesity was defined as a BMI greater than 29.9 kg/m^2^ [33].

### 2.9. Clinical Characteristics-Cardiometabolic Disease/Risk Factors

The medical history data of the patients was collected through a comprehensive questionnaire, which encompassed various cardiovascular risk factors and pre-existing conditions. Participants were asked to provide details about any previously diagnosed cardiometabolic conditions, including hypertension, type 2 diabetes, type 1 diabetes, hypercholesterolemia, elevated triglycerides, obesity, coronary heart disease, stroke, and non-alcoholic fatty liver disease. In addition, for each of these diseases, participants were also asked to provide detailed information about their current medication regimen, including the number of prescribed medicines and frequency of administration. Finally, participants were also asked about their smoking habits, alcohol consumption, and physical activity level.

### 2.10. Medication Adherence

In this study, we employed the four-item Morisky Medication Adherence General Scale [34,35], consisting of four questions with yes/no response options. The scale generates a score ranging from 0 to 4, with the developers proposing three levels of medication adherence based on this score: high adherence (0 points), medium adherence (1 to 2 points), and low adherence (3 to 4 points [34,35]). Additionally, a dichotomous definition of adherence based on the MGLS is frequently utilized, where 0 points indicate perfect adherence and 1+ points suggest some degree of nonadherence [35,36]. It is noted that the appropriate permission was requested by Fernandez-Lazaro et al. [35].

### 2.11. General Views and Beliefs Regarding the Therapeutic Protocol

Regarding the patients’ general views and beliefs about their therapeutic protocol, participants were asked to indicate their level of agreement (Agree/I am not sure/Disagree) with a set of 10 statements, comprising both negative and positive perspectives:

Negative statements

Doctors prescribe too many medicines.

People taking medicines should stop the therapeutic protocol every once in a while.

Medicines perform more harm than good.

Most medicines are addictive.

Alternative therapies are better than drugs.

I prefer to take medicines rather than restrict my diet and lifestyle.

I use alternative treatments (herbal medicines or supplements) without informing my doctor.

Positive statements

I am willing to change my habits rather than take medications.

Both the use of medicines and lifestyle changes are necessary for an effective treatment.

I believe that changes in my lifestyle play an important role in the course of my health.

For the negative statements, participants’ responses were assigned codes: Agree (−1), I am not sure (0), and Disagree (1). Conversely, for the positive statements, their responses were reverse-coded. The scores from each item were summed to yield a total scale score, ranging from −7 to 10. Higher scores reflected stronger positive beliefs in the represented concepts, whereas lower scores indicated stronger negative beliefs. Based on these scores, patients were subsequently categorized into the following two groups: Group I–Negative views/ beliefs (score: −7 to 6) and Group II–Positive views/ beliefs (score: 7 to 10).

### 2.12. Statistical Analysis

The patients’ categorical characteristics are reported as absolute frequencies (N) and relative frequencies (%), while continuous characteristics are presented as mean values with a standard deviation (SD). The normality of the continuous variables’ distribution was assessed using graphical methods (histograms, PP-plots, and QQ-plots) and the Shapiro–Wilk test.

To explore the association between the patients’ continuous characteristics and their level of adherence to medications (Perfect adherence/ Poor adherence), the Independent samples *t*-test was employed. The independent samples *t*-test was used in order to investigate the association between the patients’ continuous characteristics and their level of adherence to medications (Perfect adherence/Some level of non-adherence), while the Pearson Chi-square test was used in the case of the patients’ categorical characteristics as well as for examining the association between the level of adherence and the patients’ views/beliefs towards their therapeutic protocol. Similarly, the Pearson Chi-square test was used to examine the association between the positiveness level of patients’ beliefs/views and their demographic, socioeconomic, and clinical characteristics. To investigate the association between the level of adherence to medication and patients’ beliefs/views toward treatment, a multivariable binomial logistic regression analysis was performed. The results are presented as Odds Ratios (OR) with corresponding 95% Confidence Intervals (CI). The logistic regression models were adjusted for patients’ demographic (age, sex), socioeconomic (educational level), and clinical characteristics (number of chronic conditions they suffer from). All statistical analyses were conducted using SPSS v29.0, and the significance level (*p*-value) was set at <0.05 for two-tailed tests.

## 3. Results

### 3.1. Patients’ Demographic, Socioeconomic and Clinical Characteristics

Table 1 presents the patients’ socio-demographic and clinical characteristics, both in total as well as stratified by their level of adherence to medication. The results show that 59.4% of the patients were female, with a mean age of 63.9 (SD 13.4) years. Nearly all participants (98.6%) held Greek nationality, and 82.4% had completed at least a secondary level of education. Concerning their clinical profile, hypertension was the most prevalent condition (57.4%), followed by hypercholesterolemia (37%), and Type II diabetes (18.6%). Furthermore, 51.2% of the patients exhibited perfect medication adherence, whereas among the remaining patients (N = 970) with poor adherence, 87% showed moderate adherence (score: 1 to 2 points), and 13% demonstrated low medication adherence (score: 3 to 4 points). Moreover, as shown, individuals classified as poor adherents exhibited a lower educational level (*p* < 0.001) and a higher unemployment rate (*p* < 0.001). Additionally, among those with poor adherence, a significantly larger proportion were diagnosed with hypertension (*p* < 0.001), hypercholesterolemia (*p* < 0.001), elevated triglyceride levels (*p* < 0.001), and non-alcoholic fatty liver disease (*p* = 0.001).

### 3.2. Patients’ Beliefs/Views about Their Prescribed Therapeutic Protocol

**Figure 1:**
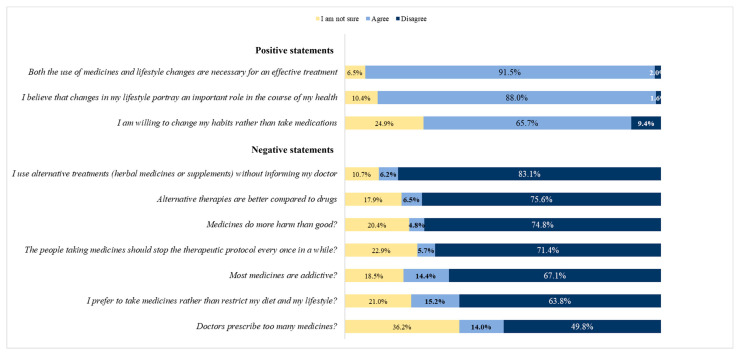
Distribution of cardiometabolic patients according to their beliefs and views regarding their treatment.

Figure 1 illustrates the patients’ beliefs/views regarding their medication. It depicts that nearly half of the patients hold either a belief or a neutral stance that their physicians prescribe an excessive number of medications. Meanwhile, 14.4% of the patients express the belief that the medication they receive can result in addictive behaviors. Only 6% of the patients believe that alternative therapies are superior to conventional drugs, and, interestingly, some of them tend to use alternative treatments without informing their physician.

Table 2 displays the patients’ beliefs/views about medication, categorized by their level of medication adherence. Among patients who were not adhering to their medication, a significantly higher percentage reported that their physician prescribed more medications than necessary (*p* < 0.001). Similarly, among non-adherers, a significantly higher percentage supported the idea that people taking medicines should stop the therapeutic protocol periodically (*p* = 0.010). Additionally, among non-adherers, a significantly higher percentage believed that alternative therapies were superior to drugs (*p* = 0.014), and a twofold higher percentage of patients preferred to take medicines rather than make dietary and lifestyle adjustments (*p* < 0.001). On the other hand, among patients with perfect adherence to medication, a significantly higher percentage expressed a willingness to change their habits instead of relying solely on medications (*p* < 0.001). Furthermore, compared to non-adherers, a significantly higher percentage of adherers reported that changes in their lifestyle play a significant role in their health’s progress (*p* = 0.023).

In Table 3, the patients’ socio-demographic and clinical characteristics are presented, categorized based on their beliefs towards medication. The findings indicate that patients with negative beliefs towards their medication tended to be older (*p* < 0.001) and were more likely to be female (*p* = 0.030). Furthermore, this group had lower levels of education (*p* < 0.001) and a higher proportion of individuals who were unemployed or retired (*p* < 0.001), as well as a higher percentage of those who were single, widowed, or divorced (*p* < 0.001). Additionally, it is worth noting that among patients with negative beliefs towards their medication, a significantly larger proportion were the patients with the highest disease burden, i.e., established coronary heart disease accompanied by a full impaired metabolic profile (all *p*-values < 0.05).

### 3.3. Impact of Patients’ Beliefs on Level of Medication Adherence

Table 4 displays the results of the multiple logistic regression analysis, examining the relationship between patients’ beliefs about medication and their level of adherence to medication. The findings reveal that patients with negative beliefs were approximately three times more likely to be non-adherent to their medication (OR = 2.73; 95% CI = 2.28–3.28). Among the specific views assessed, the belief that lifestyle changes play a crucial role in health outcomes showed the most significant impact. Patients who disagreed with this statement had at least a twofold increase in odds of being non-adherent (OR = 2.34; 95% CI = 1.10–4.97). Similarly, patients who preferred taking medications over making lifestyle changes had 2.25 times higher odds of being non-adherent (OR = 2.25; 95% CI = 1.75–2.91). Nevertheless, it is noteworthy that these patients exhibit a propensity of at least twofold to endorse the notion of medication-induced dependence while concurrently displaying approximately threefold increased odds of believing in the efficacy of interrupting medication at intervals for optimal bodily response to medication. Additionally, this group manifests twofold elevated odds of supporting the perspective that physicians often overprescribe medications. Furthermore, patients supporting the superiority of alternative therapies (OR = 1.57; 95% CI = 1.09–2.25), those believing that the medication protocol should be interrupted periodically (OR = 1.66; 95% CI = 1.13–2.44), and those who thought that physicians prescribed too many medications (OR = 1.60; 95% CI = 1.24–2.07) had at least 50% higher odds of being non-adherent compared to their counterparts.

Moreover, a stratified analysis was performed to assess the impact of patients’ beliefs towards medication on the level of medication adherence. As presented in Table 5, the investigated association was retained among female patients (OR = 2.51; 95% CI = 1.98–3.17) but not among male patients (OR = 1.27; 95% CI = 0.87–1.85). Interestingly, the effect of patients’ beliefs was more pronounced among those who had completed the secondary level of education (OR = 3.14; 95% CI = 2.06–4.77). Furthermore, the association between patients’ beliefs and the level of medication adherence demonstrated variations across different subgroups. Among employed/freelance patients, the association was stronger (OR = 2.15; 95% CI = 1.43–3.23), as well as among those who were single, widowed, or divorced (OR = 2.08; 95% CI = 1.52–2.84). Furthermore, a more pronounced link between beliefs and adherence was observed among patients with an annual income below 18,000 euros. In this income bracket, individuals holding negative beliefs/views about their prescribed medication exhibited odds of non-adherence that were at least two times higher (OR = 2.23; 95% CI = 1.50–3.32).

In terms of patients’ clinical attributes, the investigation brought to light a significant revelation. Among those who had received diagnoses of established cardiovascular diseases (coronary heart disease and stroke), there was a distinct pattern among individuals harboring negative beliefs/views regarding medication. Specifically, in this subset of patients, those holding negative beliefs/views about their prescribed medication exhibited odds of non-adherence that were almost four times higher (OR = 3.42; 95% CI = 2.32–5.05). In contrast, for patients lacking established cardiovascular diseases, the examined association did not achieve statistical significance (*p* = 0.198). Nonetheless, it is crucial to highlight that within this patient group, the impact of negative beliefs/views toward medication was found to be statistically significant (OR = 1.91; 95% CI = 1.11–3.30) among those grappling with a minimum of three distinct cardiometabolic conditions (such as hypertension, type II diabetes, hypercholesterolemia, hypertriglyceridemia, obesity, and non-alcoholic fatty liver disease).

## 4. Discussion

This study’s focus on the Mediterranean, particularly in Greece, offers valuable insights into how these regional factors intersect with patients’ beliefs, influencing their adherence to medications. This comprehension is vital for crafting interventions that align with the region’s culture and context, ultimately bolstering the effectiveness of strategies aimed at enhancing medication adherence and overall health outcomes [37]. To the best of our knowledge, this is one of the very few studies in the Mediterranean region and the first one conducted in Greece that investigated the relationship between patients’ beliefs towards medication and their medication adherence behavior in a population of cardiometabolic patients. Approximately 50% of the patients exhibited ideal adherence to their prescribed medication. This adherence trend was more pronounced in individuals with elevated educational levels, lower rates of unemployment, and fewer concurrent medical conditions. In particular, the findings revealed that a considerable proportion of patients exhibited negative views/beliefs towards their medication, with concerns regarding excessive medication use and the addictive nature of medicines. Notably, patients with negative beliefs were found to have approximately three times higher odds of being poor adherents to their prescribed therapies. Among the particular beliefs examined, patients who leaned towards choosing medications over altering their diet and lifestyle, those inclined to adjust habits instead of relying solely on medications, those advocating for alternative therapies, and those believing in the periodic interruption of medication protocol displayed lower adherence rates to medication. Conversely, stronger adherence to medication was observed among patients who believed that lifestyle changes played a pivotal role in their health trajectory, as well as among those open to habit change instead of medication reliance. Based on the present study, it is underscored that tailoring educational interventions to address patients’ negative beliefs/views or doubts about medications can be instrumental in promoting better adherence while targeting specific subgroups that could effectively address the adherence challenges experienced by these segments of the population.

### 4.1. Non-Adherence Levels among Patients with CVD and/or Cardiometabolic Disorders

Regarding medication adherence, the current study findings indicated that patients with poor adherence had lower levels of education and a higher unemployment rate. It is well established in the literature that patients with lower educational attainment may face challenges related to health literacy and understanding the significance of adhering to prescribed medications [38,39]. Limited health literacy has been linked to suboptimal medication adherence in individuals with chronic diseases, leading to inadequate management of their health conditions [40,41,42]. Similarly, individuals facing unemployment challenges may experience difficulties accessing healthcare services and medications, leading to reduced adherence. Numerous articles have established a strong association between unemployment and limited access to healthcare, as unemployed individuals often lack health insurance, leading to reduced healthcare accessibility [43]. This situation is also evident in Greece, as highlighted by Kyriopoulos et al.’s study [44], which reported that unemployed individuals with chronic illnesses are more likely to face economic barriers and encounter waiting lists, further hindering their access to essential healthcare services.

### 4.2. Importance of Patients’ Beliefs/Views

The findings of this study are in line with the existing literature regarding the impact of patients’ beliefs on medication adherence in the context of cardiometabolic conditions. Similar to previous research by Rajpura and Nayak [45] and Sjölander et al. [46], our study highlights the significant role of patients’ beliefs in shaping medication adherence. Rajpura and Nayak also reported that negative beliefs about illness and a higher perceived illness burden were associated with lower medication adherence, while positive beliefs about medications were crucial for adherence among elderly hypertensive individuals [45]. Likewise, Sjölander et al. found that beliefs about medicines exhibited stronger associations with adherence compared to illness beliefs, with non-adherent patients scoring lower on positive beliefs about medications and higher on negative beliefs [46]. Other studies have also identified beliefs about medication, concerns regarding side effects, and inadequate information provision as important barriers to medication adherence [47]. Additionally, research by Kronish et al. identified concerns about medication and knowledge of stroke prevention therapies as significant barriers among stroke survivors [48].

In particular, the findings of the present study emphasize that patients expressing concerns about their medications, including doubts about drug efficacy or a preference for alternative therapies, are more likely to display non-adherence to their prescribed medications. This alignment with previous research is evident in the work of King-Shier et al., who similarly revealed that patients may be discouraged from taking prescribed medication due to safety concerns [49]. Several other studies, encompassing both qualitative [50,51,52,53,54,55] and quantitative approaches [56,57,58], have consistently reported similar findings, highlighting that a belief in medication efficacy encourages adherence, while concerns about medication side effects and dependence are among the primary reasons for non-adherence. Additionally, our study revealed a significant association between a higher preference for alternative medications and lower medication adherence. This observation aligns with the studies of Alfian et al. [59] and Adib-Hajbaghery [60], which also demonstrated that the use of alternative medicine decreases adherence to medical medication in patients with Type II diabetes.

These findings could be attributed to the fact that patients may have reservations about the effectiveness of their prescribed medications, leading to skepticism and hesitancy in adhering to the treatment plan [61]. Furthermore, patients may also experience apprehension over potential discomfort or adverse reactions from the medications, thereby leading them to deviate from the prescribed regimen. When patients lack confidence in the medications’ efficacy and benefits in alleviating their symptoms, it can diminish their motivation to adhere diligently to the prescribed medication [62]. In addition, patients who believe in the superiority of alternative therapies, such as herbal remedies or complementary medicine, may opt for these approaches instead of following conventional medical treatments. Their preference for alternative therapies could lead to lower adherence to prescribed medications.

Furthermore, the discernible trend of enhanced medication adherence among patients valuing lifestyle changes for their overall well-being and those favoring the adoption of new habits over exclusive reliance on medications could be clarified by delving into the underlying psychological motivations of patient behavior. Patients who recognize the pivotal role of modifying their lifestyle to manage health conditions are inclined to adopt a more all-encompassing treatment approach, encompassing both medication and positive lifestyle adjustments. This comprehensive outlook is likely to yield improved treatment and medication outcomes, acting as a motivator for patients to adhere to their medication regimens. Similarly, patients receptive to altering habits might possess a proactive and engaged stance regarding their health, rendering them more likely to adhere to medication schedules and treatment strategies. Their readiness to embrace change might signify an overarching dedication to health and well-being, subsequently amplifying adherence to prescribed medications.

Additionally, the analysis of patient’s clinical characteristics within this study unveiled a significant trend, particularly among individuals grappling with established cardiovascular diseases such as coronary heart disease and stroke. Specifically, those who held negative beliefs and views regarding their prescribed medications exhibited a striking pattern. This observed pattern may be indicative of the profound psychological and emotional challenges faced by individuals dealing with significant cardiovascular conditions [63,64]. This encompasses the considerable burden of illness they carry, potential concerns related to medication side effects, and the intricate interplay of multiple health conditions that can further exacerbate their negative beliefs and doubts concerning medication efficacy. These factors collectively contribute to discouraging adherence to prescribed medications. Moreover, within this subgroup of patients with established cardiovascular diseases, negative beliefs surrounding medication can give rise to heightened fear and anxiety. Patients diagnosed with conditions such as coronary heart disease and stroke typically live with a constant awareness of the potentially severe consequences of their ailments, including the looming risk of heart attacks or strokes [65]. This heightened awareness significantly amplifies their levels of fear and anxiety, which in turn are intertwined with their negative beliefs and collectively forge a formidable psychological barrier to medication adherence. As a consequence, patients may resort to avoidance behaviors, including skipping doses or discontinuing medications altogether, as mechanisms to cope with their anxieties surrounding their health and prescribed medications [66].

It is worth noting, however, that among patients without established cardiovascular diseases, the observed relationship did not achieve statistical significance, suggesting that other factors might play a more prominent role in shaping their adherence behavior. Furthermore, among those with multiple cardiometabolic conditions, the impact of negative beliefs about medication remained significant, highlighting the intricate interaction between patients’ beliefs and their health complexity. An additional plausible interpretation of the findings within the subset of patients with established CVD and those grappling with multiple cardiometabolic conditions may relate to the phenomenon of polypharmacy. Polypharmacy, denoting the simultaneous usage of numerous medications, is a multifaceted occurrence frequently observed among individuals managing various chronic health conditions, particularly cardiovascular diseases and cardiometabolic disorders. Polypharmacy brings forth complexities such as intricate medication regimens, potential interactions between drugs, and the possibility of adverse effects [67,68]. These challenges could contribute to patients’ negative views about their prescribed medications.

Anyway, the association between patients’ beliefs and medication adherence was found to vary based on their income level, highlighting an important socio-economic dimension to the relationship. Notably, patients with an annual income below 18,000 euros exhibited a stronger connection between their beliefs and adherence behavior. Those who held negative beliefs/views about their prescribed medication within this income category were notably more likely to be non-adherent, with odds of non-adherence at least two times higher compared to their counterparts. This finding emphasizes the role of economic considerations in shaping adherence patterns [12,16]. Patients with lower incomes might face financial constraints that impact their access to medications, and negative beliefs about medication efficacy or safety could exacerbate these challenges, leading to suboptimal adherence.

## 5. Limitations

The present study has several limitations that should be acknowledged. First, this study design relied on cross-sectional data, which limits the ability to establish causal relationships between patients’ beliefs and medication adherence. Longitudinal or interventional studies would be valuable in determining the temporal sequence and potential causality of these associations. Secondly, medication adherence was assessed through self-reported measures, which may be subject to recall bias or social desirability bias. Objective measures of adherence, such as pharmacy refill records or electronic pill monitoring, could provide more accurate and reliable data on medication-taking behavior. Despite these limitations, the present study sheds light on the importance of patients’ beliefs/views in shaping medication adherence among individuals with cardiometabolic conditions. The findings pave the way for future research endeavors to delve deeper into the mechanisms underlying these associations and develop innovative interventions that can effectively address barriers to adherence, thereby enhancing patient outcomes and overall healthcare quality. By addressing these limitations and exploring new avenues of investigation, researchers can contribute to the ongoing efforts to optimize medication adherence and improve the management of cardiometabolic diseases.

## 6. Conclusions

In conclusion, this cross-sectional study has shed new light on the intricate relationship between patient beliefs and medication adherence among individuals with established cardiovascular diseases and cardiometabolic disorders within the distinct context of the Mediterranean region, with a focus on Greece. This study’s findings underscore the vital role of addressing patient beliefs as a key component of interventions aimed at promoting medication adherence. By recognizing and responding to negative beliefs, healthcare providers can forge a stronger patient-provider alliance, refine communication strategies, and tailor interventions that align with patients’ values and preferences. This study’s innovative exploration of specific negative beliefs contributes to a more nuanced understanding of the multifaceted nature of adherence behaviors. Importantly, this research demonstrates the powerful interplay of cultural, socioeconomic, and clinical factors inherent to the Mediterranean region, emphasizing the need for regionally sensitive interventions. Ultimately, the insights from this study call for the development and implementation of contextually informed strategies that can improve medication adherence and consequently enhance health outcomes in this distinct regional context.

## Figures and Tables

**Table 1:** Demographic, socioeconomic, and clinical characteristics of the N= 1988 cardiometabolic patients, stratified by their level of adherence to medication.

	Total Sample (N = 1988)	Perfect Adherence (N = 1018)	Poor Adherence (N = 970)	*p*-Value
Demographic and Socioeconomic Characteristics				
Nationality				
Greek	1961 (98.6)	1012 (99.4)	949 (97.8)	**0.002**
Other	27 (1.4)	6 (0.6)	21 (2.2)	
Sex				
Male	808 (40.6)	406 (39.9)	402 (41.4)	0.479
Female	1180 (59.4)	612 (60.1)	568 (58.6)	
Age [Mean (SD); years]	63.9 (13.4)	64.2 (12.9)	63.5 (13.9)	0.213
Level of education				
Primary education	351 (17.7)	161 (15.8)	190 (19.6)	**0.001**
Secondary education	985 (49.6)	545 (53.5)	440 (45.4)	
Higher- Tertiary education	651 (32.8)	312 (30.6)	339 (35.0)	
Occupation				
Employed/Freelance	1225 (61.6)	692 (68.0)	533 (54.9)	**<0.001**
Unemployed/Retired	763 (38.4)	326 (32.0)	437 (45.1)	
Marital status				
Married/ Cohabitation	1328 (66.8)	709 (69.6)	619 (63.8)	**0.006**
Other (Single, Widowed, Divorced)	660 (33.2)	309 (30.4)	351 (36.2)	
Annual income				
Less than 18,000 euros	1402 (70.5)	748 (73.5)	654 (67.4)	**0.003**
More than 18,000 euros	586 (29.5)	270 (26.5)	316 (32.6)	
Clinical characteristics				
Body Mass Index [Mean (SD); kg/m^2^]	27.8 (4.0)	27.8 (4.0)	27.9 (4.1)	0.411
Have you been diagnosed with:				
Coronary artery disease	352 (17.7)	178 (17.5)	174 (17.9)	0.791
Stroke	126 (6.3)	71 (7.0)	55 (5.7)	0.233
Hypertension	1141 (57.4)	518 (50.9)	623 (64.2)	**<0.001**
Type II diabetes	370 (18.6)	179 (17.6)	191 (19.7)	0.228
Type I diabetes	64 (3.2)	33 (3.2)	31 (3.2)	0.954
Hypercholesterolaemia	735 (37.0)	260 (25.5)	475 (49.0)	**<0.001**
Hypertriglyceridemia	354 (17.8)	150 (14.7)	204 (21.0)	**<0.001**
Obesity	251 (12.6)	117 (11.5)	134 (13.8)	0.119
Non- alcoholic Fatty liver disease	115 (5.8)	42 (4.1)	73 (7.5)	**0.001**
Kidney Disease	120 (6.0)	69 (6.8)	51 (5.3)	0.155

Notes: *p*-value was based on the Independent samples *t*-test (in case of continuous characteristics) and the Pearson Chi-square test (in case of categorical characteristics); The four-item Morisky Medication Adherence General Scale was used in order to evaluate the level of adherence to medication (Perfect adherence: 0 points, Poor adherence: 1+ points).

**Table 2:** Cardiometabolic patients’ beliefs and views towards treatment are stratified by their level of adherence to medication.

		Perfect Adherence (N = 1018)	Poor Adherence (N = 970)	*p*-Value
Doctors prescribe too many medicines?	Agree	114 (11.2)	163 (16.8)	**<0.001**
	I am not sure/Disagree	904 (88.8)	807 (83.2)	
The people taking medicines should stop the therapeutic protocol every once in a while?	Agree	45 (4.4)	69 (7.1)	**0.010**
	I am not sure/Disagree	973 (95.6)	901 (92.9)	
Medicines perform more harm than good?	Agree	50 (4.9)	46 (4.7)	0.860
	I am not sure/Disagree	968 (95.1)	924 (95.3)	
Most medicines are addictive?	Agree	134 (13.2)	152 (15.7)	0.111
	I am not sure/Disagree	884 (86.8)	818 (84.3)	
Alternative therapies are better compared to drugs	Agree	53 (5.2)	77 (7.9)	**0.014**
	I am not sure/Disagree	965 (94.8)	893 (92.1)	
I prefer to take medicines rather than restrict my diet and my lifestyle?	Agree	104 (10.2)	198 (20.4)	**<0.001**
	I am not sure/Disagree	914 (89.8)	772 (79.6)	
I am willing to change my habits rather than take medications	Disagree	72 (7.1)	114 (11.8)	**<0.001**
	I am not sure/Agree	946 (92.9)	856 (88.2)	
Both the use of medicines and lifestyle changes are necessary for an effective treatment	Disagree	19 (1.9)	19 (2.0)	0.881
	I am not sure/Agree	999 (98.1)	951 (98.0)	
I believe that changes in my lifestyle portray an important role in the course of my health	Disagree	10 (1.0)	22 (2.3)	**0.023**
	I am not sure/Agree	1008 (99.0)	948 (97.7)	
I use alternative treatments (herbal medicines or supplements) without informing my doctor	Agree	55 (5.4)	68 (7.0)	0.137
	I am not sure/Disagree	963 (94.6)	902 (93.0)	

**Table 3:** Demographic, socioeconomic, and clinical characteristics of the N = 1988 cardiometabolic patients, stratified by their beliefs/ views towards treatment.

	Negative Beliefs/Views (N = 870)	Positive Beliefs/Views (N = 1118)	*p*-Value
Demographic and Socioeconomic Characteristics			
Nationality			
Greek	845 (97.1)	1116 (99.8)	**<0.001**
Other	**25 (2.9)**	2 (0.2)	
Sex			
Male	330 (37.9)	478 (42.8)	**0.030**
Female	**540 (62.1)**	640 (57.2)	
Age [Mean (SD); years]	**65 (14.7)**	63 (12.3)	**<0.001**
Level of education			
Primary education	**237 (27.3)**	114 (10.2)	**<0.001**
Secondary education	346 (39.8)	639 (57.2)	
Higher- Tertiary education	286 (32.9)	365 (32.6)	
Occupation			
Employed/Freelance	336 (38.6)	889 (79.5)	**<0.001**
Unemployed/Retired/ Student	**534 (61.4)**	229 (20.5)	
Marital status			
Married/Cohabitation	508 (58.4)	820 (73.3)	**<0.001**
Other (Single, Widowed, Divorced)	**362 (41.6)**	298 (26.7)	
Yearly income			
Less than 18,000 euros	601 (69.1)	801 (71.6)	0.213
More than 18,000 euros	269 (30.9)	317 (28.4)	
Clinical characteristics			
Body Mass Index [Mean (SD); kg/m^2^]	27.9 (4.5)	27.8 (3.6)	0.459
Have you been diagnosed with:			
Hypertension	579 (66.6)	562 (50.3)	**<0.001**
Type II diabetes	216 (24.8)	154 (13.8)	**<0.001**
Type I diabetes	41 (4.7)	23 (2.1)	**<0.001**
Hypercholesterolaemia	475 (54.6)	260 (23.3)	**<0.001**
Increased triglycerides levels	219 (25.2)	135 (12.1)	**<0.001**
Obesity	129 (14.8)	122 (10.9)	**0.009**
Coronary artery disease	175 (20.1)	177 (15.8)	**0.013**
Stroke	38 (4.4)	88 (7.9)	**0.001**
Kidney Disease	43 (4.9)	77 (6.9)	0.071
Non- alcoholic Fatty liver disease	76 (8.7)	39 (3.5)	**<0.001**

Notes: *p*-value was based on the Independent samples *t*-test (in case of continuous characteristics) and the Pearson Chi- square test (in case of categorical characteristics); Based on the answers of the patients to the individual questions, a total score was created, based on which cardiometabolic patients were classified as: Group I–Negative beliefs/ views (score: −7 to 6) and Group II–Positive beliefs/views (score: 7 to 10).

**Table 4:** Odds Ratio (OR) and 95% Confidence Interval (CI) evaluating the association between the patients’ beliefs/ views towards treatment and their level of adherence to medication.

	Odds Ratio	95% Confidence Interval	*p*-Value
Negative beliefs/ views towards treatment (Ref.: Positive)	**2.73**	**2.28–3.28**	**<0.001**
I believe that changes in my lifestyle portray an important role in the course of my health (Ref.: I am not sure/ Agree)	**2.34**	**1.10–4.97**	**0.027**
I prefer to take medicines rather than restrict my diet and my lifestyle (Ref.: I am not sure/ Disagree)	**2.25**	**1.75–2.91**	**<0.001**
I am willing to change my habits rather than take medications (Ref.: I am not sure/ Agree)	**1.75**	**1.28–2.38**	**<0.001**
The people taking medicines should stop the therapeutic protocol every once in a while (Ref.: I am not sure/ Disagree)	**1.66**	**1.13–2.44**	**0.011**
Doctors prescribe too many medicines (Ref.: I am not sure/ Disagree)	**1.60**	**1.24–2.07**	**<0.001**
Alternative therapies are better compared to drugs (Ref.: I am not sure/ Disagree)	**1.57**	**1.09–2.25**	**0.014**
I use alternative treatments (herbal medicines or supplements) without informing my doctor (Ref.: I am not sure/ Agree)	1.32	0.92–1.91	0.138
Most medicines are addictive (Ref.: I am not sure/ Disagree)	1.23	0.95–1.58	0.112
Both the use of medicines and lifestyle changes are necessary for an effective treatment (Ref.: I am not sure/ Agree)	1.05	0.55–2.00	0.881
Medicines perform more harm than good (Ref.: I am not sure/ Disagree)	0.96	0.64–1.45	0.860

Notes: Results are based on the multiple logistic regression analysis and are adjusted for cardiometabolic patients’ demographic (age, sex), socioeconomic(educational level), and clinical characteristics (number of chronic conditions they suffer from); The results are presented in such a way that they explain the association of the patients’ beliefs/ views with their likelihood of being poor adherers to their prescribed medication. The four-item Morisky Medication Adherence General Scale was used in order to evaluate the level of adherence to medication (Perfect adherence: 0 points, Poor adherence: 1+ points).

**Table 5:** Results (Odds Ratio and 95% Confidence Interval) from the stratified analysis regarding the effect of the negative beliefs/ views towards treatment on the patients’ likelihood of being poor adherers to their prescribed medication.

	Odds Ratio	95% Confidence Interval	*p*-Value
Total Sample	2.73	2.28–3.28	<0.001
Stratified by:			
Sex			
Males	1.27	0.87–1.85	0.214
Females	**2.51**	**1.98–3.17**	**<0.001**
Level of education			
Primary education	1.57	0.90–2.73	0.110
Secondary education	**3.14**	**2.06–4.77**	**<0.001**
Higher/Tertiary education	**1.46**	**1.07–1.99**	**0.017**
Occupation			
Employed/Freelance	**2.15**	**1.43–3.23**	**<0.001**
Unemployed/ Retired/ Student	**1.62**	**1.19–2.22**	**0.002**
Marital status			
Married/Cohabitation	**1.49**	**1.01–2.19**	**0.045**
Other (Single, Widowed, Divorced)	**2.08**	**1.52–2.84**	**<0.001**
Yearly income			
Less than 18,000 euros/year	**2.23**	**1.50–3.32**	**<0.001**
More than 18,000 euros/year	**1.56**	**1.12–2.16**	**0.008**
Established CVD (CHD or stroke)			
Yes	**3.42**	**2.32–5.05**	**<0.001**
No	0.75	0.48–1.16	0.198
Number of clinical risk factors *			
None	1.79	0.60–5.34	0.298
One (1)	1.00	0.54–1.86	0.993
Two (2)	1.36	0.67–2.78	0.396
At least three (3)	**1.91**	**1.11–3.30**	**0.019**

Notes: Results are based on the multiple logistic regression analysis and are adjusted for cardiometabolic patients’ demographic (age, sex), socioeconomic(educational level), and clinical characteristics (number of chronic conditions they suffer from); The results are presented in such a way that they explain the association of the patients’ beliefs/ views and their likelihood of being poor adherents to their prescribed medication. * The following clinical risk factors were considered: Hypertension, Type II diabetes, Hypercholesterolaemia, Hypertriglyceridemia, Obesity and Non-alcoholic Fatty liver disease.

## Data Availability

Data are available upon reasonable request (renakosti@uth.gr).
